# Hyperspectral dataset of pure and pesticide-coated apples for measuring the level of fertilizers used

**DOI:** 10.1016/j.dib.2023.109321

**Published:** 2023-06-16

**Authors:** S. Md. Mansoor Roomi, B. Sathya Bama, V. Puvi Lakshmi, M. Vaishnavi

**Affiliations:** Department of Electronics and Communication Engineering, Thiagarajar College of Engineering, Madurai-625015

**Keywords:** Hyperspectral Imaging, Near Infrared, Apple, fertilizer, Quality Inspection

## Abstract

This dataset provides three classes of hyperspectral images: pure, insecticide-immersed, and fungicide-immersed apples with different concentrations of fertilizers. The hyperspectral images were calibrated under white and dark correction and enhanced using contrast enhancement. In order to know the variations in the level of fertilizers used, we soaked the apples in 2 different concentrations of chemicals i.e., 1ml or 1g of fertilizer in 1 liter of water as low concentration, and 3ml or 3g of fertilizer in 1 liter of water as high concentration. The proposed dataset will help in finding the consumption level of fertilizers (pesticides) in apples.

**Specifications Table**

**Table:** 

Subject	**Agricultural Sciences-** Food Science, Hyperspectral Imaging.
Specific subject area	Hyperspectral images of Apples in order to find the concentration of fertilizers used for quality inspection.
Type of data	Images
How the data were acquired	Images were captured using high-resolution Hyperspectral camera - Pika L 400-1000 nm, a line-scan camera that covers visible and near-infrared spectral range (VNIR) under perfect lighting conditions [1]. The camera was set up under proper luminance conditions and the focus was set after white and dark response correction. The captured images were visualized using Spectronon Pro software which is used for data processing and analysis. Once the camera setup was done, the object to be captured was kept on the stage with proper focus on the object, and images were captured.
Data format	Raw Filtered
Description of data collection	The apple images were captured using a hyperspectral camera which has 900 spatial Pixels Per Line and 281 spectral channels per line. The dataset consists of 617 images. There are three major categories of images 1. Fresh apple 2. Fertilizer of low concentration (1 ml/g chemical in 1 liter water) 3. Fertilizer of high concentration (3 ml/g chemical in 1 liter water) These images were taken from various angles under luminous conditions.
Data source location	Thiagarajar College of Engineering, Madurai, India. 9.8821° N, 78.0816° E
Data accessibility	Repository name: Mendeley data Data Identification Number: DOI: 10.17632/3bhr26jffs.1 Direct URL to data (.tif format): https://data.mendeley.com/datasets/3bhr26jffs Repository name: Zenodo Data Identification Number: DOI: 10.5281/zenodo.7818714 Direct URL to data (.BIL format): https://zenodo.org/record/7818714#.ZDZSTnZBy3A

## Value of the Data

• Quality inspection of edible items is a fast-growing research field [2]. The hyperspectral images of apples immersed with different levels of fungicide and insecticide will help us analyze the spectrum of the image which in turn is used for knowing the concentration level of fertilizers used.
• Agricultural enthusiasts, consumers of apples, and people working on quality inspection can be benefitted from this data.
• The provided dataset can be used for further processing through any machine learning and deep learning algorithms to make a classification between pure and pesticide-immersed apples.
• The dataset consists of 137 images of pure apples, 100 images of apples soaked in fungicide like “Nativo” and 100 images of apples soaked in insecticide like “Monostar”. In addition to this, there are also 280 sample images which might help in future research works.
• This dataset was collected using Pika L (400-1000nm) Hyperspectral imaging camera, with which the fertilizer level can be identified using the spectrum of the image.

## 1 Objective

India being an agricultural country depends on fertilizers for the healthy growth of plants by storing the soil nutrients. Even with various advantages, chemical fertilizers when consumed in higher amounts through plants or food might cause harmful effects to human health. It is necessary to know the level of fertilizers used in the food that we consume [3]. Since apples are the most consumed fruit in India, this dataset will help in knowing the chemical level present in them.

## 2 Data Description

The whole dataset consists of images of Pure apples i.e., without any fertilizer, insecticide-immersed apples (MONOSTAR) with two categories, High and Low concentration, fungicide immersed apples (NATIVO) also with two categories, High and Low concentration. Each image is saved by default as two different files with extensions of “.BIL”, a format for storing actual pixel values of the captured images band by band for each line in a file, and “.BIL.HDR”, a header file format which gives information about the nature of the data stored. The dataset provided in Mendeley repository is in the form of “.tif” format. Respective “.BIL” files of few images in .tif format is provided in Zenodo repository.

The overall dataset is divided into three folders namely “Apple_Samples”, “Fungicide_Apple”, and “Insecticide_Apple”.

In Folder – “Fungicide_Apple”, the folder named as “DIB_AppleNativo” consists of 62 images of fresh apples which includes 11 images of damaged apples as well for better research. These apples are further immersed in Nativo (fungicide) solution. The images are named in the format A1_F1, A2_F5, A2_D1, etc. where A1, A2, A3, and so on represents the number of the apple, F represents Fresh apple and D represents damaged apple image followed by the number which represents the count of the image. The Folders “DIB_AppleNativohigh” and “DIB_AppleNativolow” consists of images of apples which are immersed in fungicide solution. The data is saved in the format A1_L1, A5_L6, A2_H1, and so on where L and H represents Low concentration and high concentration.

Similarly in “Insecticide_Apple”, the folders are named as “DIB_Applemono” which consists of 75 fresh apple images, “DIB_Applemonolow” and “DIB_Applemonohigh” contains insecticide immersed apple images in both low and high concentrations. The image name is similar to that of previous formats with an M added as a prefix which represents “Monostar”.

The folder “Apple_Samples” consists of apple images which were taken as samples before capturing the actual images. The “Nativo” folder consists of 3 folders “Fresh”, “Low” and “High” with 73 images in total with the same image name format as the previous folders. The “Monostar” folder consists of 4 folders namely “Fresh”, “Low”, “High” and “Unknown conc”. The “Fresh” folder consists of 49 images of 7 different apples. These apples are immersed in monostar solution of low and high concentration levels (1ml and 3ml of solution in 1-liter water). These are named in the format 1_1PU, 2_3PU, etc. (number of the apple_count of the apple image with suffix PU which implies “pure”). “Low” and “High” folders consist of 49 images each. The name of the images are in the format 1_1HG, 3_4HG, 2_1LW and so on where the first number represents the number of the apple followed by an underscore and the count of the image in the same category. HG and LW implies high and low concentrations. The other folder “Unknown conc” folder consists of 2 folders namely “fresh” and “pesticide_unknownconc”. The “fresh” folder consists of 30 images of 6 different pure apples which are later immersed in monostar solution of unknown concentration, whose images name are in the format APP1_PU1, APP2_PU3, etc. and the “pesticide_unknownconc” folder consists of 30 images of fertilizer mixed apple with the image name in the format APP1_PST1, APP3_PST5, etc. where APP1, APP2 till APP6 represents the apple number, PU represents “pure” and PST represents “pesticide-monostar” followed by the count of the image. Detailed information about the categories and their corresponding image count of the dataset is given in Table 1.

**Table 1:** Details about the images in every category

	Fungicide		Insecticide		
Fresh	Low Concentration	High Concentration	Low Concentration	High Concentration	
137	50	50	50	50	

Apple_Samples					

	Fungicide		Insecticide		
Fresh	Low Concentration	High Concentration	Low Concentration	High Concentration	Unknown concentration

99	24	29	49	49	30

## 3 Experimental Design, Materials and Methods

Ten fresh apples were bought from the local market in Madurai for experimental purposes. After capturing the images of all 10 pure apples, the apples were separated into two groups, one for insecticide and another for fungicide immersion.

### 3.1 Preparation of Fertilizer solution

Insecticide solutions of different concentrations were used. The insecticide used was Monostar, which is a commonly used fertilizer for apples. The active ingredient of Monostar is Monocrotophos (an alkenyl phosphate) 36% SL.  The low-concentration solution contains 1ml of fertilizer in 1 liter of water and the high-concentration solution contains 3ml of fertilizer in 1 liter of water. Each apple was immersed in the low-concentrated solution for about 20 minutes and allowed to dry for 10 minutes to remove excess moisture content after which the apples were taken for image capturing. The process is repeated for the same set of apples for high-concentrated solution and the images were captured.

Similarly, for fungicide solution, the fungicide used was Nativo, which is used as a fertilizer for various fruits in India. Nativo is a new combination fungicide containing Tebuconazole (triazole) and Trifloxystrobin (strobilurin). The low-concentration solution contains 1g of fertilizer in 1 liter of water and the high-concentration solution contains 3g of fertilizer in 1 liter of water. The apples were immersed in low concentrated solution for 20 minutes and were left to dry for 10 minutes. The images were captured for this set of apples after which they were immersed in high concentrated solution for the same interval of time before the images were captured.

### 3.2 Specifications of the camera

Resonon Pika L(400-1000nm) Hyperspectral Camera is a lightweight and compact camera which has 281 spectral channels per line, 900 spatial pixels per line with a spectral bandwidth of 2.1 nm and spectral resolution of 3.3 nm. The sensor used is of CMOS type and the captured image dimensions are of 115 × 104 × 66 mm.

### 3.3 Set up and capturing of images

Before capturing the image, it is important to check all the camera settings required. The focusing of the camera is checked by using a focusing sheet and an aspect ratio sheet. Then the dark and white response is recorded. The object is kept on the stage directly under the camera focus where the luminosity is high. The captured image can be analyzed using the software.

**Fig. 1:**
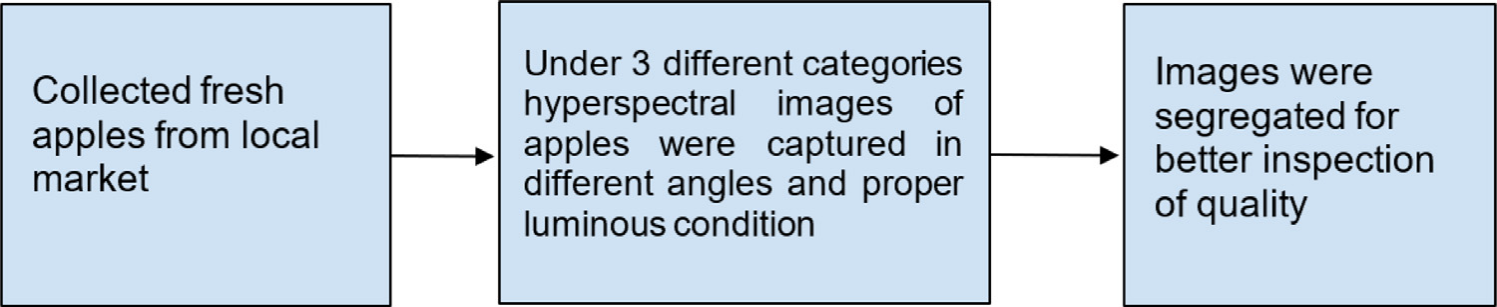
Data acquisition process.

The brief data acquisition process is given in Fig. 1.

**Fig. 2:**
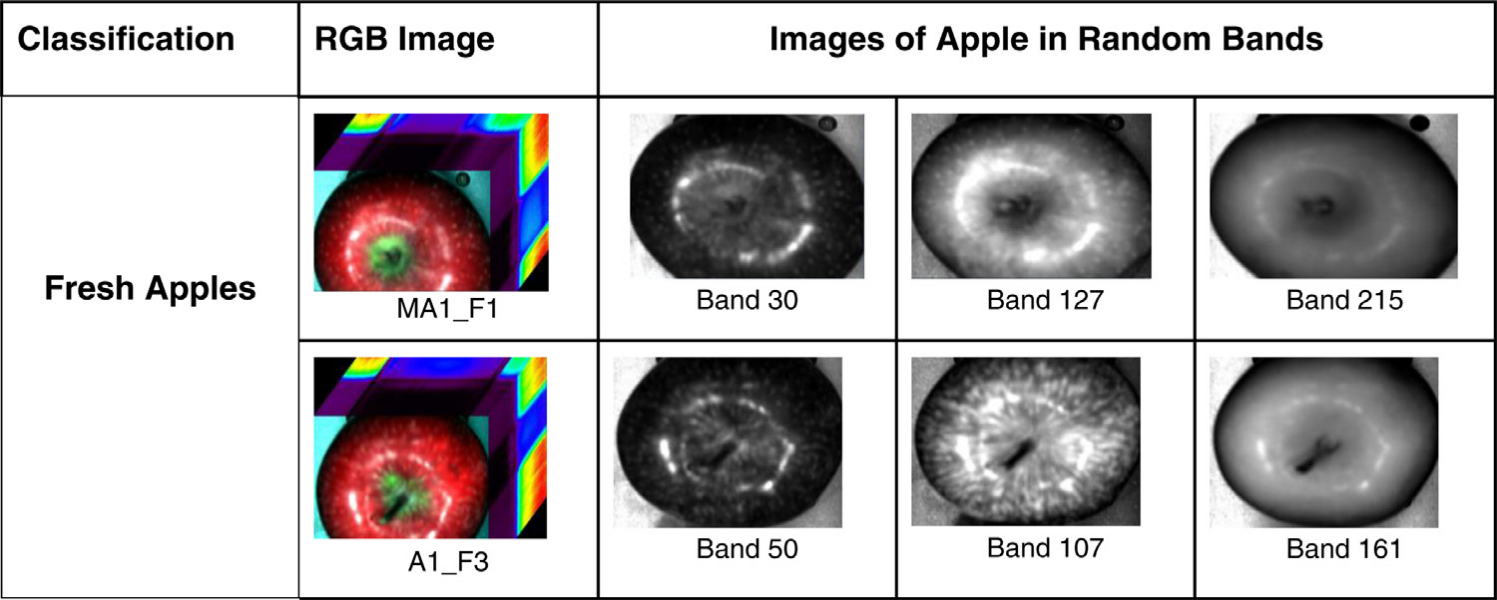
Sample Images of Apple hypercubes and randomly selected band images in .BIL format.

**Fig. 3:**
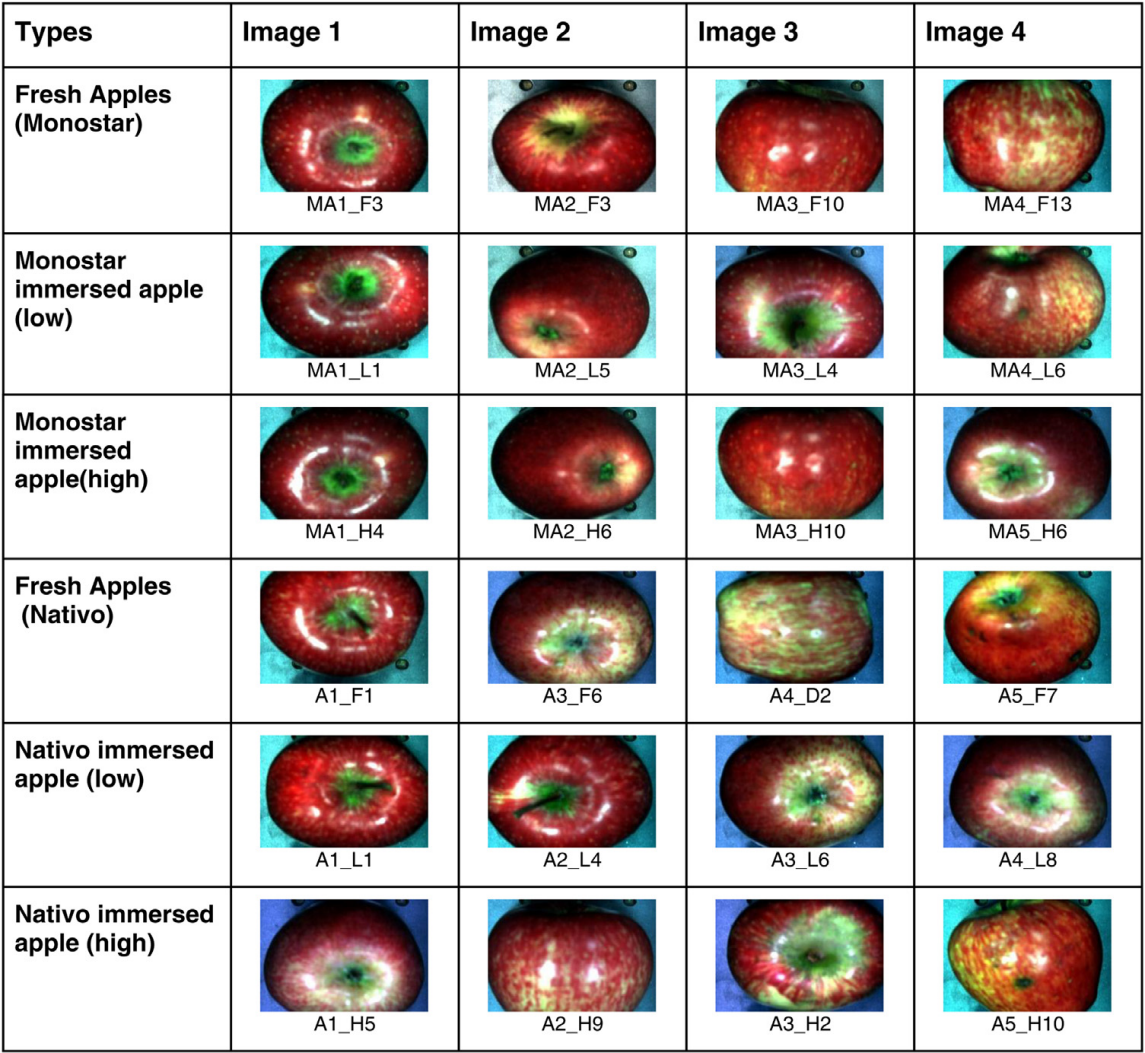
Sample Images of Apple in .tif format.

Fig. 2 represents two apple images from fresh category in various bands. This classification is possible only when the image is in “.BIL” format which is the default format for hyperspectral images. Hyperspectral images contain more than 300 spectral bands over a same spatial area which gives information about the image captured. The spectral bands shown in Fig. 2 were analyzed using the Spectronon Pro software. Fig. 3 represents the apple images in “.tif” format from each class in the proposed dataset.

## Ethics Statements

This paper does not involve any works based on human subjects and animal experiments.

## CRediT authorship contribution statement

**S. Md. Mansoor Roomi:** Conceptualization, Methodology. **B. Sathya Bama:** Conceptualization, Methodology. **V. Puvi Lakshmi:** Data curation, Writing – review & editing. **M. Vaishnavi:** Data curation, Writing – original draft, Writing – review & editing.

## Declaration of Competing Interest

The authors declare that they have no known competing financial interests or personal relationships that could have appeared to influence the work reported in this paper.

## Data Availability

Apple-Hyperspectral images (Original data) (Mendeley Data). Hyperspectral dataset of pure and pesticide-coated apples for measuring the level of fertilizers used. (Original data) (Zenodo).
